# Unravelling the role of obesity and lipids during tumor progression

**DOI:** 10.3389/fphar.2023.1163160

**Published:** 2023-03-30

**Authors:** Junzhe Zhao, Keene Lee, Han Chong Toh, Kong Peng Lam, Shi Yong Neo

**Affiliations:** ^1^ Cancer and Stem Cell Biology, Duke-NUS Medical School, Singapore, Singapore; ^2^ Division of Medical Oncology, National Cancer Centre Singapore, Singapore, Singapore; ^3^ Singapore Immunology Network (SIgN), Agency for Science, Technology and Research (A*STAR), Singapore, Singapore; ^4^ School of Biological Sciences, Nanyang Technological University, Singapore, Singapore; ^5^ Department of Microbiology and Immunology, Yong Loo Lin School of Medicine, National University of Singapore, Singapore, Singapore; ^6^ Department of Oncology and Pathology, Karolinska Institute, Solna, Sweden

**Keywords:** hepatocellular carcinoma, NASH, lipid, adipocytes, obesity

## Abstract

The dysregulation of the biochemical pathways in cancer promotes oncogenic transformations and metastatic potential. Recent studies have shed light on how obesity and altered lipid metabolism could be the driving force for tumor progression. Here, in this review, we focus on liver cancer and discuss how obesity and lipid-driven metabolic reprogramming affect tumor, immune, and stroma cells in the tumor microenvironment and, in turn, how alterations in these cells synergize to influence and contribute to tumor growth and dissemination. With increasing evidence on how obesity exacerbates inflammation and immune tolerance, we also touch upon the impact of obesity and altered lipid metabolism on tumor immune escape.

## 1 Introduction

There is accumulating evidence of a link between environmental factors and cancer incidence rates. Environmental factors related to lifestyle choices, which include high-carbohydrate and high-lipid diets, have been strongly correlated with metabolic disruptions leading to obesity, insulin resistance, and even metabolic syndrome. Obesity, a result of excessive systemic adipose tissue accumulation leading to increased body weight, is strongly associated with the development of a host of chronic diseases such as type 2 diabetes mellitus, hypertension, cardiovascular disease, and hepatic steatosis. Furthermore, it has been implicated as a risk factor in the initiation and progression of a wide variety of cancers (Dobbins et al., 2013) which include breast, ovary (Liu et al., 2015), prostate, liver, colon, and pancreatic cancers.

Cancer can be regarded as a metabolic disease. Despite obesity being a risk factor for cancer development, the scientific link between obesity and tumor progression is not apparent. In a meta-analysis of 33 studies across different cancer types, obesity is revealed as a prognostic but not an independent predictor for the survival outcomes of cancer patients (Parekh et al., 2012). Several obesity-associated molecular factors and signaling pathways such as adipokine production, alterations in the insulin-like growth factor 1 (IGF-1) signaling pathway, excessive steroidal sex hormone production, chronic low-grade inflammation, oxidative stress, and gut dysbiosis were identified to influence tumor cell biology, but the underlying mechanisms have not been fully elucidated (Parekh et al., 2012; Park et al., 2014a; Avgerinos et al., 2019).

**FIGURE 1:**
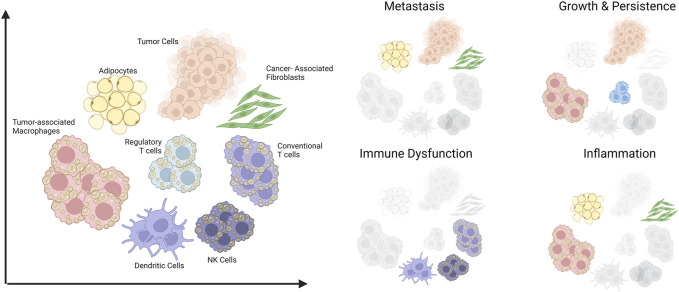
Schematic summary of cells in the tumor microenvironment that are amenable to lipid-induced metabolic programming and their contributions to aspects of cancer progression. The scope of this review covers various cell types within the tumor microenvironment **(Left)** and their involvement in four key aspects of tumor progression **(Right)**. Created with BioRender.com.

In this review, we cover how dysregulated lipid metabolism could fuel cancer progression. Using hepatic steatosis and liver cancer as a classic example, we discuss how obesity could lead to metabolic disease and also oncogenesis. Finally, we will also highlight the role of lipids as a metabolic immune checkpoint in the stroma and immune compartments of the tumor microenvironment (TME) (Figure 1).

## 2 Altered lipid metabolism in cancer

**FIGURE 2:**
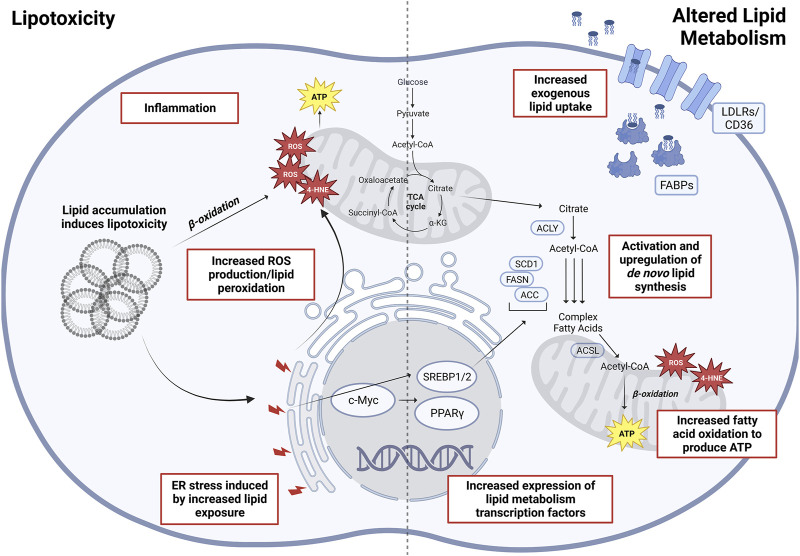
Altered lipid metabolism and lipotoxicity in oncogenesis. Depicted **(Left)** are various factors associated with lipid accumulation and are lipotoxicity induced, eventually increasing overall cellular stress leading to oncogenesis and malignant transformations. **(Right)** Main altered pathways in lipid metabolism associated with cancers. Abbreviations: ROS, reactive oxygen species; 4-HNE, 4-hydroxynonenal; SREBP1/2, sterol regulatory element–binding proteins 1/2; PPARγ, peroxisome proliferator-activated receptor gamma; ACLY, ATP-citrate lyase; SCD1, stearoyl-CoA desaturase 1; ACSL, long-chain fatty acid–CoA ligase 1; FASN, fatty acid synthase; ACC, acetyl-CoA carboxylase; FABP, fatty acid–binding proteins; LDLR, low-density lipoprotein receptor. Created with BioRender.com.

Given that lipids play an integral role in cellular metabolism (Carracedo et al., 2013), signaling, and membrane structure, altered lipid metabolism has been regarded as one of the more important metabolic alterations associated with tumor cell growth and cancer progression (Rohrig and Schulze, 2016). A systematic analysis of omics data from TCGA has revealed that fatty acids (FAs), arachidonic acid (AA), and cholesterol metabolism, together with peroxisome proliferator-activated receptor (PPAR) signaling came up as the top hits of altered lipid metabolism pathways (Hao et al., 2019). Furthermore, the pathways involving altered lipid uptake, storage, recycling, and *de novo* synthesis have been associated with cancer progression and metastasis (reviewed in detail in Mehla and Singh, 2019; Fu et al., 2020; Vasseur and Guillaumond, 2022), making lipid metabolism enzymes potentially attractive targets for cancer treatments (Figure 2).

The oncogenic activation of *de novo* synthesis of cholesterol and FAs would allow tumor cells to rely less on exogenous uptake of lipids (Pizer et al., 1996; Roongta et al., 2011; Menendez et al., 2016). Multiple enzymes within the FA synthesis pathway which include ATP-citrate lyase (ACLY) (Bauer et al., 2005; Hatzivassiliou et al., 2005), acetyl-CoA carboxylases (ACC1/2) (Jones et al., 2017; Ye et al., 2019), FA synthase (FASN) (Flavin et al., 2010; Fhu and Ali, 2020), and stearoyl-CoA desaturase (SCD1) (Roongta et al., 2011; Mason et al., 2012; Chen et al., 2016; Luis et al., 2021) have all been found to correlate strongly with tumor growth and have been proposed as biomarkers for specific types of cancers. Modulating expression levels of these enzymes are transcription factors from the SREBP family (SREBP1/2), interacting with or modulated by key oncogenic signaling nodes which include the PI3K/AKT signaling axis (Ricoult et al., 2016; Yi et al., 2020) and c-Myc (Wu et al., 2016). Importantly, SREBP1/2 upregulations have been observed in several cancers (Bao et al., 2016; Ricoult et al., 2016), making these potential therapeutic targets that globally downregulate *de novo* lipid synthesis. Another transcription factor PPARγ controls the expression levels of the mediators of lipid metabolism, playing important roles in regulating FA oxidation (FAO), FA storage, and cholesterogenesis (Grygiel-Gorniak, 2014). Its downregulation has been implicated in a worse prognosis in non–small-cell lung cancer (Sasaki et al., 2002), while its higher expression is correlated with increased survival in colorectal cancer (CRC) patients (Ogino et al., 2009) and reported as a favorable prognosis marker in breast cancer (Abduljabbar et al., 2015). Conversely, tumors could at the same time often upregulate the expression of lipid uptake receptors (Kuemmerle et al., 2011). In particular, the upregulation of fatty acid transporter CD36 has been associated with many different cancer types such as oral, breast, ovarian, cervical, and gastric cancers (Pascual et al., 2017; Luo et al., 2021). Furthermore, CD36 overexpression has been associated with faster tumor growth, cancer progression, and metastasis initiation (Pascual et al., 2017; Luo et al., 2021; Guerrero-Rodriguez et al., 2022; Ruan et al., 2022).

Increases in exogenous uptake and *de novo* synthesis of lipids have to be complemented by concomitant increases in lipolysis, particularly to capitalize on the increased availability of lipids as a bioenergetic source. Consequently, FAO has been observed as another metabolic adaptation in tumor cells that is necessary for continued tumor growth and metastasis (aside from aerobic glycolysis and increased reliance on glutaminolysis), especially under unfavorable and possibly poorly vascularized and hypoxic tumor microenvironments (Carracedo et al., 2013; Ma et al., 2018a). Shifts in FAO reliance for the production of ATP and reducing metabolites such as NADPH, classified as a “lipolytic phenotype” (Ma et al., 2018a), can be attributed to the dysregulation of expression levels of key FAO genes such as CD36 (discussed above), carnitine palmitoyltransferase I/II (CPT1/2) (Xiong et al., 2020), carnitine transporter 2 (CT2) (Wang et al., 2021), and various classes of acyl-CoA synthetase long-chain family members (ACSLs) (Padanad et al., 2016). This lipolytic phenotype provides the metabolic flexibility of cancer cells to increase lipid availability, whether it be for energy production, membrane synthesis, or cell signaling. The reliance on FAO in cancer cells has been shown to be necessary for tumor proliferation, survival, maintenance of stemness, drug resistance, and even metastatic progression, as reviewed by Ma et al. (2018a). Considering that tumor cells could rely more on fatty acid oxidation to fuel their metabolic needs, an *in vitro* study found that dampening lipid metabolism with etomoxir treatment results in reduced metastatic potential and growth arrest of bladder cancer cells (Cheng et al., 2019). Notably, metastatic cells within lymph nodes are protected by oleic acid within the tumor microenvironment, conferring resistance to oxidative stress and ferroptosis that enable them to subsequently form secondary tumors (Ubellacker et al., 2020). Taken together, the various alterations to lipid metabolism in cancers is clearly integral to multiple cellular and signaling processes involved in continued tumor survival and cancer progression.

## 3 NAFLD/NASH-HCC

Altered lipid metabolism has a significant influence in the development of non-alcoholic fatty liver disease (NAFLD), non-alcoholic steatohepatitis (NASH), and even hepatocellular carcinoma (HCC). Fatty liver disease (hepatic steatosis) is the hallmark of NAFLD/NASH and commonly the first step in the tumorigenesis cascade. Epidemiology shows that one in four in the world’s population has NAFLD at some point in their life and, alarmingly, the prevalence of this global epidemic has risen by almost 8% in the past 5 years (Younossi et al., 2019; Riazi et al., 2022). NASH is a more severe form of NAFLD, where steatosis is accompanied by inflammation and hepatocellular ballooning, with or without fibrosis (Chalasani et al., 2018). NAFLD/NASH represents a significant risk factor for HCC where 10%–20% of NAFLD/NASH patients may eventually develop HCC, overtaking viral hepatitis as the leading HCC etiology globally (Huang et al., 2021). Cancers like HCC are not simply driven by oncogenic mutations or malignancy emerged from chronic inflammation, HCC entails considerable metabolic reprogramming such as altered lipid metabolism (Anstee et al., 2019). Gross histopathology examination of resected HCC tumors often shows irregular nodules that are more yellow and soft than the adjacent normal (Schlageter et al., 2014), indicating steatosis and altered lipid metabolism. A recent characterization of the NASH-HCC transcriptome shows apparent upregulation of the pathways involved in FA metabolism (Pinyol et al., 2021). The complex metabolic and inflammatory aberrations lead to increased proliferation of hepatobiliary progenitors (Gadd et al., 2014), deranged genomic and epigenomic stability (Nishida et al., 2016), and escape from immune surveillance (Pfister et al., 2021), which ultimately drive tumorigenesis.

The development of NAFLD/NASH follows the natural trajectory of disease progression, and hyperinsulinemia (an indication of insulin resistance) correlates with HCC incidence (Chettouh et al., 2015). A high fat diet or high intake of carbohydrates and calories, in general, can lead to insulin resistance and subsequently to NAFLD/NASH or even metabolic syndrome. Insulin resistance sits at the center of metabolic syndrome, comprising abdominal obesity, hyperglycemia, dyslipidemia, and hypertension—the co-occurrence of which significantly predisposes to 2 diabetes (T2DM) and atherosclerotic cardiovascular diseases (ASCVD). While the exact causal relationships between insulin resistance and increased HCC risk is not fully understood, insulin resistance leads to increased circulating free FAs and glucose, as well as *de novo* lipogenesis in the hepatocytes (Smith et al., 2020). It comes as no surprise that drugs that increase insulin sensitivity, such as metformin, can improve NAFLD/NASH histology and subsequently reduce the risk of HCC (Plaz Torres et al., 2022). Synergistically, deranged free cholesterols could potentially lead to malignant progression. The accumulation of free cholesterols may be due to increased *de novo* synthesis (Simonen et al., 2011; Min et al., 2012), as there is a high substrate load and upregulation of HMG-CoA reductase. There seems to be a decreased absorption of plasma cholesterol (Simonen et al., 2011) and decreased LDL receptor expression (Min et al., 2012) by the hepatocytes, which may explain the dyslipidemia and increased ASCVD risk. To add on, a high fructose diet is also notorious for its effect in driving NAFLD/NASH. Fructose drives *de novo* lipogenesis, suppresses fatty acid oxidation, and damages mitochondrial integrity in hepatocytes, hence increasing lipid accumulation and hepatocellular damage (Yu et al., 2021a). Interestingly, genetic predisposition has been indicated in the development of lipotoxicity and hepatic steatosis. *PNPLA3* polymorphism or mutation is probably the best-known genetic predisposition to NAFLD/NASH, fibrosis, and HCC (Liu et al., 2014; Dong, 2019). *PNPLA3* encodes a triglyceride lipase that often binds to intracellular lipid droplets. The altered forms of PNPLA3 protein lead to impaired lipid metabolism and hence, increased lipid droplet accumulation, resulting in lipotoxicity and downstream cellular stresses (Dong, 2019). Experiments have also shown a seemingly genetic predisposition of NAFLD/NASH when abnormal hepatic differentiation is observed from patient-derived iPSCs when compared with those from healthy individuals (Kimura et al., 2022).

Further understanding of the molecular basis of NAFLD/NASH has proven to be critical to better design potential therapeutic interventions. Several transcription factors, enzymes, and transporters have been implicated in the accumulation of lipid *via* dysregulation of processes such as *de novo* lipogenesis, circulating free FAs (e.g., from diet), and adipolysis. FXR agonists (e.g., obeticholic acid) (Neuschwander-Tetri et al., 2015) and PPARγ agonists (e.g., pioglitazone) (Chalasani et al., 2018) have been shown to improve NAFLD/NASH histology, with both targets regulating lipid uptake and metabolism. Sterol response element binding protein 1c (SREBP-1c) is also a central transcription factor controlling cellular FA metabolism (Wang et al., 2015). Induced by insulin, it increases the expression of acetyl-CoA carboxylase (ACC) (Lally et al., 2019) and fatty acid synthase (FASN) (Jones and Infante, 2015), hence upregulating the production of malonyl-CoA and FAs. SREBP-1c is upregulated in the hepatocytes during insulin resistance, NAFLD/NASH, and HCC (Williams et al., 2013), indicating that *de novo* lipogenesis represents an indispensable source of lipid accumulation. In addition, interstitial FAs are transported into hepatocytes by fatty acid binding protein 1 (FABP1), lipid scavenger receptors, and fatty acid transport proteins (FATPs), depending on the length of the FAs. These carrier proteins may directly influence lipid accumulation and hence accelerate disease progression. While silencing of *FABP1* directly ameliorates hepatic steatosis (Mukai et al., 2017), its effect on HCC development seems to depend more on increased secretion of vascular endothelial growth factor-A (VEGF-A) (Ku et al., 2016), therefore promoting angiogenesis. Of note, one of the scavenger receptor, CD36, is significantly expressed in higher levels in HCC than in adjacent normal tissues (Nath et al., 2015), which leads to increased FA uptake (Nath et al., 2015) and activates the Warburg effect *via* the mTOR/PI3K/AKT pathways (Luo et al., 2021).

Apart from the bioenergetics fueled by alterations in lipid metabolism, lipotoxicity could lead to intrinsic cellular stress that may also drive the oncogenic progression of NAFLD/NASH to HCC (Figure 2). Increased substrate loading, mitochondrial metabolic activity, and inflammation inevitably led to increased production of reactive oxygen species (ROS) and products of lipid peroxidation (Begriche et al., 2013; Anstee et al., 2019). The exhaustion of cellular antioxidant mechanisms in turn causes increased lipid accumulation, forming a vicious cycle. It is also well known that increased oxidative stress can lead to DNA damage and genomic/epigenomic instability, which is more prominent in NAFLD/NASH patients and those who have progressed with HCC (Tanaka et al., 2013; Nishida et al., 2016). Likewise, studies have elucidated the cross talk between endoplasmic reticulum (ER) stress and lipid metabolism. Lipid exposure leads to increased ER stress in hepatocytes and HCC cells (Cao et al., 2012), which in turn causes increased ROS production and hepatocellular damage. The *MUP-uPA* mouse model of NASH-HCC relies on both a high fat diet and the overexpression and ER accumulation of urokinase plasminogen activator (uPA) in the hepatocytes for tumorigenesis (Febbraio et al., 2019). ER stress also modulates lipid metabolism by activating SREBP-1c and causing oxidative stress (Egnatchik et al., 2014; Han and Kaufman, 2016). Such cross talk complements the tumorigenic role of TNF upregulation and inflammation induced by ER stress (Nakagawa et al., 2014). In addition, the accumulation of cholesterols also contributes to ER stress and mitochondrial dysfunction, resulting in hepatocellular damage and inflammation (Malhotra et al., 2020; Zhou and Sun, 2021). While the above discussion of hepatic steatosis focuses primarily on hepatocyte metabolism, the complexity of NAFLD/NASH and HCC involve an extensive remodeling of the liver microenvironment with lipid-driven fibrosis and chronic inflammation. Within the stroma, cholesterol can trigger the metabolic reprogramming of Kupffer cells (KCs) and hepatic stellate cells (HSCs) which in turn activate these cells into a pro-inflammatory state (Arguello et al., 2015). Subsequent sections of the review will further discuss the effects of lipid dysregulation on adipocytes, cancer-associated fibroblasts, immune myeloid cells, and lymphocytes.

## 4 Role of adipocytes in tumor development

Within the lipid-laden tumor microenvironment, the involvement of adipocytes could in part explain the association between obesity and cancer progression. Of note, adipocytes produce pro-inflammatory cytokines such as IL-6, IL-8, and TNFα (Bruun et al., 2003; Ritchie and Connell, 2007), and angiogenic factors such as VEGF, prostaglandins and leukotrienes, which not only have pro-tumorigenic correlations but have been associated with cancer progression, metastasis, and even immune evasion (Wang and DuBois, 2016; Tian et al., 2020). With obesity, the accumulation of adipocytes within the breast tumor microenvironment could also result in high collagen IV production implicating chronic inflammation and tissue fibrosis (Park and Scherer, 2012). Inhibition of hypoxia-inducible factor 1α (HIF1α) was demonstrated to prevent the activation of adipocytes leading to reduced fibrosis and inflammation during obesity (Sun et al., 2013). It was also shown that excessive adipocyte accumulation, particularly in visceral areas around tumor initiation sites, can induce a hypoxic microenvironment (Park et al., 2014b), promoting angiogenesis and contributing to cancer metastasis.

Cancer cells can secrete factors that modulate adipocyte FA metabolism, forming a reciprocal interaction which enhances the availability of FA, fueling cancer growth (Lengyel et al., 2018). For example, breast cancer cells can secrete exosomes containing pro-lipolytic factors such as miR-144 and miR-126, which stimulate lipolysis within neighboring adipocytes through the activation of AMPK and autophagy. As a result, enhanced lipolysis and the release of FAs shift the metabolic dependencies of migrating cancer cells toward increasing exogenous FA uptake and their reliance on β-oxidation for energy supply (Balaban et al., 2017). Likewise, inflammatory cytokines secreted by adipocytes were found to enhance the metastatic potential of breast cancer cells (Dirat et al., 2011). Interestingly, multiple cancers such as renal, gastric, breast and colon cancers have been shown to preferentially grow in adipocyte-rich environments. Furthermore, metastatic prostate,breast, and ovarian cancer cells preferentially home toward adipocyte tissues in the vicinity, including peri-glandular regions and the visceral omentum.

Conversely, the activation of brown adipose tissue (BAT) may have opposing roles that are beneficial to the tumor-bearing host. A recent study has demonstrated that the UCP1-dependent activation of brown adipose tissue dampens the glycolytic pathways within cancer cells to influence the growth of several cancer types as demonstrated in mouse models and a human study (Seki et al., 2022). Indeed, our current understanding is that there is an interesting functional plasticity of adipocytes in their involvement during cancer progression. Yet, the role of adipocytes remains underappreciated in the field and should be further studied in specific cancers.

## 5 Lipid metabolism and cancer-associated fibroblasts

An emerging cell type that has been revealed to heavily influence tumor progression and metastasis within the TME is cancer-associated fibroblasts (CAFs). These cells are known to modulate the TME through various mechanisms (reviewed by Sahai et al., 2020; Ping et al., 2021) which include, but are not limited to 1) extracellular matrix remodeling, 2) immune cross talk through chemokine and cytokine production, 3) soluble factor secretions which include angiogenic and growth factors (VEGF and HGF), microRNAs (in exosomes), and lastly, the most relevant to this discussion, 4) metabolic modulation through lactate, alanine, and aspartate shuttling and amino acid depletion (Wu et al., 2017). Apart from carbohydrate and amino acid modulations within the TME, CAFs are also known to modulate the bioavailability of lipids both directly and indirectly to fuel the metabolism of cancer cells within the often nutrient-starved TME. Of note, Santi et al. (2015) have revealed the direct transfer of lipids from CAFs to cancer cells *via* ectosomes, fueling cancer growth. Gong et al. (2020) also confirmed lipidomic reprogramming of CAFs in CRC having enhanced *de novo* FA synthesis and reduced lipid catabolism phenotype. The study further reveals FASN upregulation in CAFs, which together with CD36 upregulation in CRC cells, promotes this intimate metabolic cross talk and facilitates both CRC cell migration and growth. A complementary study done in lung cancer revealed that CAF-derived lipids, particularly oleic acid, can activate lipid metabolism in cancer cells *via* the upregulation of SCD1 under glucose-starved conditions. Furthermore, oleic acid transfer from CAFs could enhance cancer cell stemness (Hwang et al., 2022). Further examples of metabolic reprogramming occurring in CAFs to facilitate increased lipid availability and transfer to cancer cells include the role of pancreatic stellate cells, which upon activation, releases a wide range of lipids, such as lysophosphatidylcholines (LPCs). These LPCs go on to enhance phosphatidylcholine synthesis in pancreatic ductal adenocarcinoma (PDAC) cells, activating wound healing mediators which enhance PDAC cell proliferation and migration (Auciello et al., 2019). The emerging roles of CAFs as a lipid source fueling cancer cell growth, proliferation, and metastasis place emphasis on the requirement for further studies to dissect lipid cross talk between CAFs and cancer cells and present an additional interface of cancer therapeutic design.

## 6 Metabolic reprogramming of myeloid cells in cancer

### 6.1 Macrophages

Different macrophage populations possess distinct influences on homeostasis and disease beyond phagocytosis, as discovered by Elie Metchnikoff (PMID: 27477126). In 2004, the M1-M2 spectrum of macrophage polarization was proposed (Mantovani et al., 2004; Mantovani et al., 2013), and the concept of tumor-associated macrophages (TAMs) was subsequently pioneered (Mantovani et al., 2017). Indeed, macrophages can be a “friend” or “foe” in almost every step of pathogenesis and disease progression for a vast number of diseases (Mantovani et al., 2022; Wculek et al., 2022). A recent study has demonstrated that CAF reprogramming of blood monocytes converts them into lipid-associated macrophages with an immune suppressive phenotype. These macrophages are STAB1^+^ and TREM2^high^ and reported to be expanded in non-responders of immune checkpoint therapy (Timperi et al., 2022).

A reciprocal relationship exists between macrophage phenotype and metabolism. On one hand, the macrophage activation pattern leads to its metabolic reprogramming. M1 macrophages adopt a glycolytic metabolic profile downstream of LPS/IFN-γ activation of toll-like receptors (TLRs) and interferon gamma receptors, along with increased induced nitric oxide synthase (iNOS) activity (Viola et al., 2019; Liu et al., 2021; Wculek et al., 2022). This is to facilitate the rapid turnover of metabolites during phagocytosis and production of pro-inflammatory cytokines and NO. FAs are rapidly synthesized and deployed for eicosanoid synthesis, while fatty acid oxidation (FAO) is blocked (Batista-Gonzalez et al., 2019; Yan and Horng, 2020). Together with lipid overload and triglyceride synthesis, there is increased intracellular lipid accumulation in the M1 state (Xiang et al., 2018; Rosas-Ballina et al., 2020; Morgan et al., 2021). By contrast, M2 macrophages activated by IL-4 primarily utilize oxidative phosphorylation (OXPHOS) and FAO (Viola et al., 2019; Wu et al., 2020; Liu et al., 2021; Wculek et al., 2022). IL-4 stimulation activates STAT6, which in turn increases the expression of lysosomal acid lipase (LAL) and scavenger receptors like CD36 (Huang et al., 2014) and MARCO (Georgoudaki et al., 2016), upregulating lipoprotein/FA uptake and lipolysis. However, FAO is not required for M2 polarization (Nomura et al., 2016), suggesting compensatory metabolic pathways that may be induced by AMPK or PPARγ (Namgaladze and Brune, 2016).

On the other hand, metabolic derangements can alter macrophage phenotype, which depends on the local environment which includes its biochemical (metabolic) environment (Mehla and Singh, 2019). The overload of saturated fatty acids (SFAs) can directly bind to TLRs and cause M1 polarization (Namgaladze and Brune, 2016). The increased accumulation of lipids (Heymann et al., 2015), ROS (Yan and Horng, 2020), and lactic acid (Colegio et al., 2014) will in turn cause cellular damage and death, causing further M1 activation. However, a lipid-/lactate-rich environment can cause both a transient “burst” of M1 polarization as well as the selection of M2 for survival (Camell and Smith, 2013; Colegio et al., 2014), which may explain the discrepancy between a pro-inflammatory secretome and an anti-inflammatory cellular profile. High glucose (Pavlou et al., 2018) and fructose (Yu et al., 2021a) exposure may also shift macrophage phenotypes. While it causes an increase in both pro-inflammatory cytokines in M1 and anti-inflammatory cytokines in M2, there is also an aberrant macrophage metabolism with reduced glycolytic capacity, implying a decrease in phagocytosis and NO production (Pavlou et al., 2018). Abnormal cholesterol metabolism has also been indicated in different disease settings but generally leads to pro-inflammatory macrophage phenotypes (Malhotra et al., 2020; Morgan et al., 2021). Finally, hypoxia may alter macrophage functioning as well, yet the precise action remains elusive (Sadiku and Walmsley, 2019).

It has been shown that macrophages *in vivo* adopt a spectrum of pro- and anti-inflammatory phenotypes, where a dichotomy of M1 *versus* M2 is an oversimplification (Liu et al., 2021). Indeed, much evidence has suggested that macrophages can possess surface markers classically defined for both M1 and M2, and their phenotypes contribute to disease progression or improvement in a spatiotemporally dependent manner. In cancer, it is recognized that these global biochemical and metabolic aberrations generally lead to a pro-inflammatory polarization at the pro-tumor necroinflammation stage, and an anti-inflammatory polarization in the full-blown tumor (Lee-Rueckert et al., 2022). The same also applies to ASCVD development (Lee-Rueckert et al., 2022), where chronic inflammation leads to M1-like cholesterol ester accumulation and foam cell formation, followed by M2-driven remodeling before acute plaque rupture and thrombosis *via* inflammation again (Sukhorukov et al., 2020; Wculek et al., 2022). TAMs are subject to extreme metabolic conditions such as hypoxia, nutrient deprivation, environmental acidity, and a myriad of cytokines both pro-inflammatory (TNFα, IL-1, IL-6, and IFN-γ) and anti-inflammatory (adenosine, IL-10, and TGF-β) (Mantovani et al., 2022). Hence, it is important therapeutically that we target specific metabolic pathways at different stages of tumor development. For instance, systemic glycolytic inhibitors may reduce lactic acid–driven M2 polarization yet compromising M1 activation at the same time. Although blocking lipid uptake or FAO may result in abrogation of anti-inflammatory TAM functions (Georgoudaki et al., 2016), therapeutics targeting TAM metabolism have yet a long way to go before clinical impact (Mantovani et al., 2022). Interestingly, a recent study has shown glycolytic TAMs are the major PD-L1 expressors in HCC, and PD-L1 blockade can unleash glycolysis-dependent tumoricidal activity (Lu et al., 2022). Future studies may investigate the relationship between metabolism and immune checkpoints (Yu et al., 2021b) and design novel immuno-oncology interventions powered by metabolomics (Bleve et al., 2020).

### 6.2 Dendritic cells

The metabolic profile of dendritic cells (DCs) in cancer bears resemblance to that of macrophages. DC activation causes a metabolic switch from OXPHOS to glycolysis (Krawczyk et al., 2010; Williford et al., 2019), with increasing lipid accumulation that may facilitate antigen presentation (Bougneres et al., 2009). However, the influence of metabolic dysregulation on DC phenotype is more prominent in tumors. The antigen processing and presentation in lipid-laden DCs are compromised, resulting in defective T-cell stimulation (Herber et al., 2010). Metabolic reprogramming of DCs from glycolysis to FAO depends on PPARγ, conferring an immunosuppressive effect similar to M2 activation (Zhao et al., 2018). Lipid peroxidation and accumulation can also lead to ER stress (Cubillos-Ruiz et al., 2015) and decreased antigen processing at the ER (Veglia et al., 2017), abrogating antitumor immunity. While neoantigen-based DC vaccine is a promising immuno-oncology strategy, it may be necessary to interrogate the extracellular or intracellular biochemical environment to avoid the negative impact by metabolic derangements on DC functions (Wculek et al., 2019; Moller et al., 2022). The use of a carnitine palmitoyltransferase-1 (CPT1) mitochondrial fatty acid transporter inhibitor—etomoxir—could be a promising candidate for combinatory therapy with conventional immune checkpoint inhibitors where the metabolic reprogramming of DCs can be abrogated to prevent polarization into a tolerogenic phenotyping that drives the generation of regulatory T cells (Zhao et al., 2018).

### 6.3 Myeloid-derived suppressor cells

Myeloid-derived suppressor cells (MDSCs) are a heterogeneous group of immunosuppressive myeloid cells that are differentiated in chronic inflammatory conditions such as cancer (Hegde et al., 2021). These cells may not possess typical granulocyte or monocyte/macrophage phenotypes and hence a monolithic view of MDSCs can see a dichotomy of polymorphonuclear (PMN) and monocyte (M) MDSCs. The current understanding has uncovered much heterogeneity of MDSCs and an alternative framework has been proposed (Hegde et al., 2021). MDSCs reprogram their metabolism in ways resembling immunosuppressive macrophages and dysfunctional DCs. MDSCs upregulate FA uptake and oxidation (Al-Khami et al., 2017), which is abrogated by FAO inhibitors that delay tumor growth and synergize with other therapies (Hossain et al., 2015). Evidence suggests that this process is mediated by inflammation, hypoxia, and environmental acidity, which in turn upregulate HIF-1α (Corzo et al., 2010), PPARγ (Xin et al., 2021), AMPK (Yan et al., 2019), and STAT3 (Ma et al., 2018b). Increased eicosanoid metabolism (Gabrilovich, 2017), characterized by increased COX-2 activity and concentrations of AA and prostaglandin E2 (Prima et al., 2017; Wang et al., 2022), reciprocally increases lipid accumulation and confers immunosuppressive activity. The nitrogen metabolism is more complex, as increase in both arginase-1 (Raber et al., 2012), a potent T-cell suppressor, and iNOS (Enioutina et al., 2011), the pro-inflammatory NO synthase, has been reported. The immunosuppressive effect is a delicate balance of mTOR/PI3K/AKT regulation as well as stimulation by Th1 or Th2 cytokines (Yan et al., 2019). Finally, ER stress may also confer MDSC immunosuppression *via* the PERK-NRF2 pathway (Mohamed et al., 2020). Ablating PERK, a kinase on the ER, upregulates type I interferon response and reprograms the MDSCs toward tumor-limiting myeloid cells (Mohamed et al., 2020). Taken together, metabolic reprogramming of MDSCs lead to an increased expression of myeloid checkpoints [e.g., PD-L1 (Prima et al., 2017; Xin et al., 2021) and arginase-1 (Grzywa et al., 2020)] and immunosuppressive cytokines [e.g., TGF-β (Gabrilovich, 2017), IL-10 (Grzywa et al., 2020), and PGE_2_ (Wang et al., 2022)]. Targeting MDSC metabolism, such as lipid transport (Al-Khami et al., 2016)/FAO inhibitors (Hossain et al., 2015; Xin et al., 2021), LXR agonists (Tavazoie et al., 2018), and COX-2 inhibitors (Prima et al., 2017), may synergize with immune checkpoint blockade and confer clinical benefit.

## 7 Obesity-mediated reprogramming of tumor-infiltrating lymphocytes

Studies in recent years have started to shed light on the link between obesity and antitumor immune responses. With an increasing focus on immunotherapy in cancer, there is a greater need to understand how altered lipid metabolism would influence the performance of cytotoxic lymphocytes within the tumor microenvironment.

### 7.1 T cells

A systematic analysis has revealed that an abundance of CD8 T cells was the most predictive parameter of a beneficial response to anti-PD-1/PD-L1 therapy across multiple cancer types (Lee and Ruppin, 2019). As exemplified in mouse models, a high-fat diet resulted in poor CD8 T-cell infiltration into several types of solid tumors. Colorectal tumors of patients with higher BMI of more than 35 were also associated with lower CD8 immune scores (Ringel et al., 2020). Our current understanding is that the shift toward lipid metabolism drives cellular stress and T-cell dysfunction. In fact, it has been elucidated that PD-1 ligation elevated FAO in T cells during immune suppression which can be modulated by conventional immune checkpoint blockade (Patsoukis et al., 2015). In addition, accumulation of LCFAs dampens mitochondrial respiratory capacity and triggers extensive transcriptional reprogramming of CD8 T cells (Manzo et al., 2020). Considering that the bioenergetics of intra-tumoral T-cells have shifted toward FAO rather than glycolysis, the activation of PPARγ enables beneficial metabolic adaptation that boosts mitochondrial functions and oxidative phosphorylation despite the altered lipid metabolism (Chowdhury et al., 2018).

As a scavenger receptor for LCFAs and oxidized LDL, CD36 is highlighted in recent studies as an emerging target for cancer therapeutics. It has been demonstrated that a high-fat diet enhances the potential of CD36^+^ tumor cells to initiate the formation of metastases (Pascual et al., 2017). Being both a signal transducer and lipid transporter, the expression of CD36 influences the activation of both conventional and regulatory T cells, implying that CD36 could be a potential metabolic immune checkpoint to target. The deletion of CD36 would reduce the uptake of oxidized lipids that is associated with ferroptosis and impaired CD8 T-effector functions. At the same time, CD36-triggered AMPK and PPARγ signaling could also be suppressive pathways that are distinct from metabolic stress (Chen et al., 2022). Unlike conventional T cells, regulatory T cells are programmed to express lower GLUT-1 with a higher reliance on lipid oxidation (Michalek et al., 2011). It has been demonstrated that CD36 mediates altered lipid metabolism that enables PPARβ-mediated adaptation to high lactate environment for the survival of regulatory T cells (Wang et al., 2020).

### 7.2 Natural killer cells

Similar to T cells, obesity has also been recently reported to influence natural killer (NK) cell–mediated antitumor immunity. In a comparison of normal weight and obese human subjects, it was found that the frequency of NKG2D^+^ and CD56^dim^ cytotoxic NK cells in the peripheral blood was reduced (Bahr et al., 2018). Reduced frequencies of NK cells in the spleen and liver were also reported in obese mice which would potentially increase the risk of cancer development (Bahr et al., 2017). Lipotoxicity impairs mTOR activation in NK cells and glycolytic activity which are both essential for its effector functions. Scavenger receptors such as CD36, CD68, and MSR1 were found upregulated in dysfunctional NK cells which were reported to be induced by granulocytic MDSCs (Niavarani et al., 2019). Mechanistically, lipid-treated NK cells acquire defects in lytic granule polarization despite successfully forming an immune synapse with its tumor target (Michelet et al., 2018). Likewise, etomoxir was also reported to restore NK cell functionality under lipid stress (Michelet et al., 2018). While we previously reported that NK cells could experience cumulative oxidative stress in lung cancer patients with smoking history, it was also found that obesity increases the susceptibility of NK cells to the harmful effects of cigarette smoke (Yang et al., 2020; O’Shea et al., 2010). Obesity not only has suppressive effects on NK cell cytotoxicity but also influences the immune-regulatory functions of NK cells. An increased proportion of a NK cell subset expressing IL6Ra was observed in obese mice and humans in which transcriptomics analysis revealed an enrichment of inflammatory genes that is suggestive that these regulatory NK cells could contribute to obesity-associated inflammation (Theurich et al., 2017).

## 8 Concluding remarks

So far, the oncogenic role of obesity on the tumor-bearing host has been mostly studied in HCC where hepatic steatosis is a prominent driver of malignancy. Despite the existing vast literature on altered lipid metabolism, most studies largely focus on characterizing the metabolic changes that reprograms the bioenergetics of tumor cells. Within the gut–liver axis, the obesity-augmented microbiome and metabolites may have significant influences on the host’s immune responses during tumor progression. A recent study has reported differential bile acids and gut microbiome profiles comparing HCC responders *versus* non-responders to immune checkpoint blockade (Lee et al., 2022). Additionally, the gut microbiome influences levels of short-chain fatty acids that influence the functional plasticity of hepatic immune cells (Hu et al., 2023). Given that obesity is often implicated in chronic inflammation, future studies should seek to understand how obesity influences the systemic immune responses of the tumor-bearing host. Ultimately, these novel insights on metabolism could change existing treatment and lifestyle modification paradigms to provide the next breakthroughs in oncology.
